# Splenic Calcification in a Case of Sarcoidosis

**DOI:** 10.31138/mjr.32.3.276

**Published:** 2021-09-30

**Authors:** Prasanta Padhan, Debashis Maikap

**Affiliations:** Department of Clinical Immunology and Rheumatology, Kalinga Institute of Medical Sciences, KIIT University, Bhubaneswar, Odisha, India

## CASE REPORT

**Figure 1.**
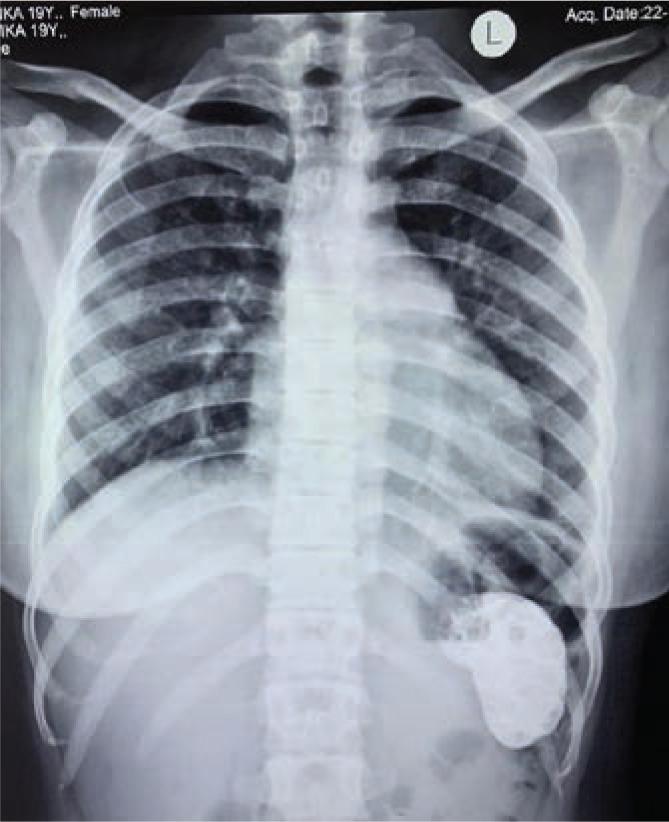
Chest X ray (PA view) showing calcified atrophic spleen.

A 19-year-old girl presented to us with history of low-grade fever and recurrent painful nodular lesions in both legs for 3 months duration. She also complained of bilateral ankle pain and swelling of 2 weeks duration. She had no similar history in the past and her family history was unremarkable. On examination she was febrile with a temperature of 100° F, and lower limb examination revealed bilateral erythema nodosum and ankle arthritis. Other systems examination was normal. Her investigations revealed raised ESR, 78mm in 1^st^ hour and raised C reactive protein of 26mg/L (Normal < 6mg/L). Her complete blood count, liver function and renal function tests were normal. She had raised serum calcium, 11.5mg/dL. She had negative Mantoux and TB Quantiferon test. Her serum angiotensin converting enzyme (ACE) level was 96U/L (Normal range 16–85 U/L). She had serum Vitamin D3 level of 40ng/mL (Normal range 20–30ng/mL).She had normal ferritin and serum immunoglobulin levels and negative serology for hepatitis B, and C and HIV. Serology for brucellosis and histoplasmosis were negative. Her chest radiograph showed features of splenic calcification in the left hypochondrium (**Figure 1**). Computed tomography of thorax showed bilateral hilar adenopathy and splenic calcifications. A diagnosis sarcoidosis was made. She was started on oral prednisolone 40mg per day along with methotrexate at a dose of 15mg per week and hydroxychloroquine 200mg per day. She was vaccinated for Pneumococcus and Hemophilus influenzae. She promptly became afebrile with resolution of arthritis and skin lesions within 4 weeks. Her serum calcium normalized and her serum ACE level reduced to 56U/L. She was regularly followed up for 6 months and was off corticosteroid. She lost follow-up subsequently.

However, one year later, she presented to our emergency department with history of fever with associated productive cough of 5 days duration. She took anti-cold medication with no benefit. She got high grade intermittent fever (103°F) with chill and rigor for last 2 days and developed shortness of breath for last 1 day. On examination, she had altered sensorium and hypotension. Chest imaging revealed bilateral pneumonia with evolving ARDS. She resuscitated with fluid initially, followed by vasopressor. Empiric antibiotic meropenem with clarithromycin was started after blood culture. However, her general condition deteriorated in 24hr despite adequate medical management and she died of overwhelming sepsis. Subsequent blood culture report revealed Streptococcus pneumoniae.

## DISCUSSION

Sarcoidosis is a multisystem granulomatous inflammatory disease of unknown aetiology. The most commonly involved organs are the lungs and the lymphoid system. Extrapulmonary involvement of sarcoidosis is reported in 30% of patients, and the abdomen is the most common extra-thoracic site with a frequency of 50%–70%. Liver (50%–80%), spleen (40%–80%), lymph nodes (30%), and kidney are frequently involved abdominal sites, sometimes without symptoms.^1^ Cardiopulmonary involvement is the main cause of death. Granulomatous infiltration of the spleen is common in sarcoidosis and has been reported in 24%–53% of cases. Splenic infiltration can be homogeneous or in the form of multiple discrete nodules.^2^ However, splenic calcification is rare.

Splenic calcification either can be focal (non-homogenous) or diffuse. Non-homogenous or focal splenic calcifications may result from splenic haemangioma or lymphangioma, hydatid cyst, splenic hematoma (trauma), splenic infract (sickle cell disease) splenic artery aneurysm, pyogenic or tubercular abscess, hemochromatosis, and neoplasm.^3^

The diffuse splenic calcifications can be either punctate (starry spleen) or uniform. Diffuse punctate calcifications of spleen are seen in brucellosis (associated with suppurating lesions), tuberculosis, pneumocystis jirovecii (commonly associated with kidney and lymph node calcifications), candidiasis, histoplasmosis (larger lesions and usually more than six in number), sarcoidosis, and amyloidosis (along with calcifications in liver)^3,4,5^ Homogenous calcification with atrophic spleen is rarely seen in conditions such as sickle cell disease and occasionally in thorotrast administration.^3^

Although there are relatively few differential diagnoses, the pattern and the setting in which such calcification presents helps us pin-point the diagnosis. We have ruled out common causes: chronic infection such as tuberculosis, histoplasmosis, brucellosis, and sickle cell disease. Patients with sarcoidosis have been shown to have impaired vascular endothelial function and increased arterial stiffness according to a study by Siasos et al. of 87 patients with sarcoidosis.^6^ Eventually, chronic perivascular granulomatous inflammation resulting in the periarteriolar fibrosis seen on histology may have compromised vascular supply, leading to splenic ischaemia and infarction.

Radiological evidence of calcification in sarcoidosis is uncommon and it indicates chronicity and functional asplenic state. This in turn can result in fulminant sepsis due to secondary immune deficiency as in this case, in which case the mortality may be more than 50%.^7^ Hence, prompt recognition of infections and early treatment can prevent death in such individuals.

The causative organisms are usually polysaccharide-encapsulated bacteria such as Streptococcus pneumoniae, Haemophilus influenzae, and Neisseria meningitides.^7^ In our case, despite the patient having received pneumococcal vaccine (Pneumovax 23), she developed pneumococcal sepsis. This might be due to infection of other strains of pneumococci, or the fact that immunosuppression at that point failed to produce adequate immune response. Education, vaccination against this organism, and/or use of preventive antibiotics can prevent post splenectomy overwhelming infection and sepsis.
